# How Do French–English Bilinguals Pull Verb Particle Constructions Off? Factors Influencing Second Language Processing of Unfamiliar Structures at the Syntax-Semantics Interface

**DOI:** 10.3389/fpsyg.2018.01885

**Published:** 2018-10-17

**Authors:** Alexandre C. Herbay, Laura M. Gonnerman, Shari R. Baum

**Affiliations:** ^1^School of Communication Sciences and Disorders, McGill University, Montreal, QC, Canada; ^2^Centre for Research on Brain, Language and Music, Montreal, QC, Canada

**Keywords:** second language sentence processing, syntax-semantic interface, working memory, lexical knowledge, phrasal verbs, verb-particle constructions, bilingualism, native-like processing

## Abstract

An important challenge in bilingualism research is to understand the mechanisms underlying sentence processing in a second language and whether they are comparable to those underlying native processing. Here, we focus on verb-particle constructions (VPCs) that are among the most difficult elements to acquire in L2 English. The verb and the particle form a unit, which often has a non-compositional meaning (e.g., *look up* or *chew out*), making the combined structure semantically opaque. However, bilinguals with higher levels of English proficiency can develop a good knowledge of the semantic properties of VPCs (Blais and Gonnerman, 2013). A second difficulty is that in a sentence context, the particle can be shifted after the direct object of the verb (e.g., *The professor looked it up).* The processing is more challenging when the object is long (e.g., *The professor looked the student’s last name up*). This shifted structure favors syntactic processing at the expense of VPC semantic processing. We sought to determine whether or not bilinguals’ reading time (RT) patterns would be similar to those observed for native monolinguals (Gonnerman and Hayes, 2005) when reading VPCs in sentential contexts. French–English bilinguals were tested for English language proficiency, working memory and explicit VPC semantic knowledge. During a self-paced reading task, participants read 78 sentences with VPCs that varied according to parameters that influence native speakers’ reading dynamics: verb-particle transparency, particle adjacency and length of the object noun phrase (NP; 2, 3, or 5 words). RTs in a critical region that included verbs, NPs and particles were measured. Results revealed that RTs were modulated by participants’ English proficiency, with higher proficiency associated with shorter RTs. Examining participants’ explicit semantic knowledge of VPCs and working memory, only readers with more native-like knowledge of VPCs and a high working memory presented RT patterns that were similar to those of monolinguals. Therefore, given the necessary lexical and computational resources, bilingual processing of novel structures at the syntax-semantics interface follows the principles influencing native processing. The findings are in keeping with theories that postulate similar representations and processing in L1 and L2 modulated by processing difficulty.

## Introduction

Learning a second-language (L2) presents numerous challenges, leading to a considerable range of attainment levels for different speakers. Many underlying causes, whether specific to the L2 learners or to their environment, play a role in the observed differences in second language acquisition (SLA), including but not limited to: age of acquisition and cognitive factors (Granena and Long, 2013; Nicoladis and Montanari, 2016), motivation (Gao, 2010; Mady, 2014), learning strategies, type of L2 exposure (Munoz, 2008), and identification with the L1 or L2 culture (e.g., Lybeck, 2002; Hochman and Davidov, 2014). The large number of factors influencing L2 knowledge and proficiency increases the difficulty of identifying the mechanisms underlying second language sentence processing and whether the latter follows the same principles as native speakers’ processing. Indeed, this may depend not only on whether bilingual processing is similar to that of native speakers for different linguistic levels involved in sentence processing, but also on individual characteristics of the second language speakers.

Reading a sentence involves almost simultaneously orthographic, morpho-syntactic, semantic and pragmatic processes in order to recognize words, integrate them in a sentence structure, and understand its meaning in a given context. Psycholinguistic methods have proven to be efficient to investigate sentence processing in real time and evaluate whether bilinguals employ the same or different strategies than native speakers, but also to unveil subtle processing differences that might exist within bilingual individuals (Abrahamsson and Hyltenstam, 2009).

In this study, we explore the bilingual processing of Verb Particle Constructions (VPCs) that, despite being very frequent in spoken language, are among the most complex structures to acquire in L2 English. We aim to investigate if French–English bilinguals are sensitive to the same parameters as English monolinguals when processing VPCs, and what individual factors might modulate this sensitivity and the ultimate attainment that bilinguals can reach at the interface of syntax and semantics. Before considering theories of second language processing, we will first define VPCs more precisely, including formal analyses of VPCs, and how they are processed by native speakers.

### Semantic and Syntactic Properties of Verb Particle Constructions

Verb particle constructions, also known as phrasal verbs, are semantic units composed of a verb and a particle, which can be superficially similar to either a preposition or an adverb, such as *run off, chew out*, or *finish up*. There are more than 3000 VPCs in English (MacArthur and Atkins, 1974). They have two main syntactic and semantic features. First, the position of the particle is not fixed: it can be either adjacent to the verb, such as in sentences (1), (3), (5), and (7) or shifted after the object noun phrase (NP) as in (2), (4), (6), and (8).

(1) Deirdre will *finish up* her project
(2) Deirdre will *finish* her project *up*
(3) Deirdre will *finish up* her exciting project on bilingualism
(4) Deirdre will *finish* her exciting project on bilingualism *up*
(5) Deirdre will *chew out* her friend
(6) Deirdre will *chew* her friend *out*
(7) Deirdre will *chew out* her facetious friend from school
(8) Deirdre will *chew* her facetious friend from school *out*

Secondly, VPC meaning is not always a combination of the meanings of the verb and particle. Indeed, VPC transparency ranges from transparent VPCs, such as *finish up* in sentences (1–4), to very opaque VPCs that have a non-compositional meaning, such as *chew out* in sentences (5–8) (non-compositional, opaque, idiomatic are considered as synonyms here).

These two properties have led to a considerable debate about VPCs’ underlying syntactic structure and the variables affecting their processing (see Haiden, 2006, for a review). The different particle placement preferences have led some authors to argue that the VPC structure is not uniform and two main syntactic structures have been proposed. In the *complex head* approach, the verb and particle are represented as a single syntactic constituent, a complex verbal head formed either morphologically in the lexicon (Booij, 1990; Johnson, 1991; Stiebels and Wunderlich, 1994), or by an incorporation of the particle into the verb (Van Riemsdijk, 1978; Zeller, 2001). This strong link between the verb and particle would require an adjacent particle placement. In the *small clause* approach, the verb and particle are represented as separate syntactic elements (Kayne, 1985; Hoekstra, 1988; Den Dikken, 1995). The particle has its own syntactic position and can therefore be either adjacent or shifted, forming a *small clause* with the object NP. Wurmbrand (2000) suggested that transparent and opaque verbs are each reflected by a different underlying syntactic structure: opaque VPCs would be associated with the complex-head structure while transparent VPCs would be represented by the small clause structure. Punske (2013) has even suggested the existence of three structures by proposing two different kinds of small clause structures. However, Brehm and Goldrick (2017) showed that VPCs may be associated with multiple syntactic structures along a gradient, rather than with only one of two discrete categories. The current study is not intended to differentiate among the various theoretical linguistic structures posited; therefore, we remain neutral toward these proposals and focus instead on processing aspects of different surface word orders.

### Corpus Studies Exploring Factors Influencing Particle Placement

Aside from the more formal syntactic analyses, a number of studies have aimed to understand what factors would favor each particle placement in different syntactic and semantic configurations. In a corpus study, Gries (2003) examined 25 phonological, morphosyntactic, semantic and discourse variables that have been said to influence word order alternation, in order to describe, explain and predict particle placement. Only a subset of the 25 factors assessed in multifactorial analyses significantly affected particle placement, with the 5 most influential factors including NP length and complexity, VPC transparency, and the type of NP (pronominal, proper name, lexical). Gries integrated all of these significant variables under his so-called Processing Hypothesis (Gries, 1999, 2003), which states that a speaker chooses a word order to communicate the intended message in a clear way with as little processing effort as possible for both the speaker and the listener. Applied to VPCs, particle placement would therefore be determined by the processing effort that the object NP requires: adjacent particles will be preferred for VPCs with direct objects requiring a great deal of processing effort (long or complex ones) and for opaque VPCs, whereas particles will be more easily shifted when direct objects require little processing effort or when the VPC is transparent. The Processing Hypothesis successfully brings several significant factors influencing particle placement into a single framework; however, it does not provide a computational mechanism to quantify how each factor is linked to processing cost.

An alternative account of VPC processing was proposed by Hawkins (2004), following from the observation that word order and semantic relationships between words play an important role in native speakers’ ease of comprehension. According to the Performance–Grammar Correspondence Hypothesis (Hawkins, 2004), constituent length and order influence complexity of sentence structure and associated processing efficiency. Only the easiest configurations are preferred and incorporated into the grammar. Following this hypothesis, Lohse et al. (2004) aimed to integrate some of the previously-studied factors influencing particle placement, namely length and structure of the object NP and VPC transparency, into a single principle of processing efficiency: particle positions that minimize the size of various syntactic and semantic dependencies will be the easiest to process, and will therefore be preferred. In this framework, the domain associated with a dependency between X and Y is the smallest sequence of words containing X and Y and their semantic or syntactic properties necessary to process the dependency relation. Sentence configurations minimizing the size of this domain – usually minimizing the distance between X and Y – will be preferred. We will briefly describe three different relevant domains for particle placement, namely the Verbal Phrase Phrasal Combination domain, the Verb-Particle Semantic Dependency domain and the Particle-Head NP domain.

First, the Verbal Phrase Phrasal Combination domain (hereafter VP domain) follows from the Early Immediate Constituent (EIC) principle (Hawkins, 1994) which states that a word order that allows earlier access to all immediate constituents will be preferred. Thus, the VP domain is defined by the minimal sequence of words containing all the elements necessary to build a VP, namely the verb, the particle and the first element of the object NP. This domain is minimal when the particle is adjacent; indeed, for this word order, one can access the verb, the particle and the first word of the NP by reading only three words. On the other hand, for shifted particles, one would need to read the verb and the whole NP before accessing the particle; this would minimally require reading 3 words for 1-word NPs and more for longer NPs, resulting in a larger VP domain. Therefore, VP domain minimization accounts for the preference of adjacent particles when the object NP is long. In the four studied corpora in Lohse et al. (2004), the proportion of shifted particles decreased when the NP length increased: 40% of particles were shifted after 2-word NPs, 18% after 3-word NPs, and only 3% after NPs of 5 words or more.

The second domain is the Verb-Particle Semantic Dependency domain (VPC dependency domain). From a processing perspective, VPC semantic opacity is formalized as a semantic dependency between the verb and the particle. This semantic dependency is low for transparent VPCs, such as *finish up*. Indeed, the meaning of the VPC is not very dependent on the particle as it only slightly changes the meaning of the verb (*to finish up* entails *to finish*). On the contrary, the dependency is high for opaque VPCs, such as *chew out*, as the particle is needed to access the full VPC meaning, which is quite different from the meaning of the verb alone (*to chew out* does not entail *to chew*). The domain associated with the semantic dependency between the verb and the particle (hereafter VPC dependency domain) is defined by the minimum sequence of words containing the verb and the particle. It is minimized (only two-words long) when the particle is adjacent to the verb, as the reader accesses the particle immediately after the verb. For shifted particles, the VPC dependency domain also includes the intervening NP, which makes it necessarily larger than for adjacent particles. The processing pressure to minimize the VPC dependency domain is higher when the semantic dependency is stronger between the verb and the particle. In addition, because of the large semantic discrepancy that can exist between a verb and an associated high dependency VPC, such as between *chew* and *chew out*, shifted particles tend to create local incongruity at the object NP, as the latter often seems less plausible with the verb alone, before one has access to the shifted particle. For example, in sentence (5), ‘*her friend*’ seems incongruous when just reading “*Deirdre will chew her friend”* before accessing *‘out.’* Therefore, this domain minimization accounts for the preference of adjacent particles for high dependency VPCs, while there is less pressure on particle placement for low dependency VPCs. In corpora, a larger proportion of shifted particles for lower dependency VPCs than for higher dependency VPCs was found for all NP lengths (Lohse et al., 2004: Figure 2).

Finally, the last domain, the Particle–NP Head lexical dependency domain, is only relevant for low dependency VPCs and is defined by the minimum sequence of words containing the particle and the head of the object NP. When the head of the NP is closer to its beginning, this domain is minimized by adjacent particles, such as in *Deirdre will finish up her project on bilingualism*, where the Particle–NP Head is 3-words long instead of 4-words long if the particle is shifted. When the head of the NP is toward its end, shifted particles minimize Particle–NP Head domain such as in *Deirdre will finish her exciting new project up* (2 words instead of 5 words for adjacent particles). In corpora, 30% of shifted particles were found when the head of the NP was at the end instead of 13% when it was followed by a few words (Lohse et al., 2004, p. 252). The particle is less needed in low dependency VPCs as it only slightly changes the meaning of the verb. Consequently, shifted particles would be an advantage as the object NP would appear before the particle and allow an earlier access to all important elements of the sentence, namely the subject, the main verb and the object NP, without having to process the particle upfront.

Minimization of different domains can align toward a given particle placement or go in opposite directions, each domain minimization coming at the cost of another. Overall, minimizing the syntactic and semantic domains favors adjacent particles for longer NPs and more dependent VPCs. Less dependent VPCs allow for both placements, with some advantages for shifted particles for head-final NPs. To facilitate interpretation and presage the expected processing patterns anticipated for native speakers, **Table 1** illustrates the different particle placement preferences for different configurations using sentences (1) to (8) introduced earlier.

**Table 1:** Particle placement preferences according to VPC dependency and NP length.

Sentences	VPC dependency	NP length	Placement preference
(1–2)	Low	Short	Potential preference for shifted particles as NPs are very often head-final (2)
(3–4)	Low	Long	Preference for adjacent particles (3)
(5–6)	High	Short	Strong preference for adjacent particles (5)
(7–8)	High	Long	Very strong preference for adjacent particles (7)

### Experimental Studies on Factors Influencing Particle Placement

To examine processing of VPCs in monolinguals and determine whether the findings would be in keeping with predictions based on the domain minimization patterns just described, Gonnerman and Hayes (2005) assessed native English speakers’ knowledge of VPCs’ semantic dependency by measuring their sensitivity to the semantic similarity of VPCs and their corresponding verbs, either using explicit judgements (e.g., “*How similar are the meanings of look out and look on a scale from 1 to 9?*”) or in a masked priming task. Monolingual speakers were found to be sensitive to VPCs’ gradation in similarity both explicitly – their similarity judgements were accurate and consistent across speakers using the entire scale between 1 and 9 – and implicitly, as they only showed facilitation for low dependency VPCs.

A self-paced reading experiment in the same study showed that, when processing VPCs in a sentence context, English monolinguals were influenced by three factors, namely the VPCs’ semantic dependency, the particle position and the direct object NP length, with higher reading times (RTs) for sentences with high dependency VPCs, shifted particles and longer NPs. Highest reading times were found when the combination of two factors accentuated the difficulty of a particular word order: shifted particles with either long NPs or high dependency VPCs. In addition, using the same paradigm, but including different types of relative clauses inside the direct object NPs, Gonnerman (2012) showed that monolinguals were also sensitive to NP complexity, with higher reading times for sentences with shifted particles when the complexity increased. An overall Particle Position by VPC Dependency interaction was also found: sentences including low dependency VPCs were read faster when the particle was shifted (*Deirdre will finish her project up*) than when the particle was adjacent (*Deirdre will finish up her project*), while it was the opposite for high dependency VPCs for which shifted particle sentences (*Deirdre will chew her friend out*) were read more slowly than adjacent particle sentences (*Deirdre will chew out her friend*). The patterns found in monolingual native speakers of English were in line with the principles of complexity and processing efficiency proposed by Lohse et al. (2004). Now, the question is whether bilingual VPC processing follows the same principles.

### L2 Acquisition of Verb Particle Constructions

Verb-particle constructions are particularly difficult to acquire for L2 speakers. Indeed, one verb can often be associated with many particles and their meaning is often non-compositional. Bilinguals usually avoid VPCs and prefer using synonyms, regardless of the existence of such constructions in their native language (see Blais and Gonnerman, 2013, for a review on production and teaching of VPCs). Moreover, VPCs can have several meanings. Garnier and Schmitt (2016) showed that Spanish–English bilinguals knew at least one meaning of only 40% of the most frequent polysemous English VPCs, and all meanings of only 20% of these VPCs. Corpus frequency was found to predict VPC knowledge, along with time per week spent reading and on social networks.

This laborious L2 acquisition of VPCs contrasts with the absence of any specific problem associated with VPCs in L1 language development (Behrens, 1998). However, these difficulties do not seem well accounted for by some theories of Second Language Acquisition that attempt to define which level of language processing is especially difficult in a second language and would be the ‘*bottleneck’* of L2 acquisition. The Bottleneck hypothesis (Slabakova, 2008) states that functional morphology (the morphology–syntax interface) is the bottleneck of acquisition and by contrast, structures at the syntax–semantics interface are not especially difficult to acquire in an L2. Meaning would come for free if the functional morpho-syntactic competence were already in place. In the same way, the Interface Hypothesis (Sorace and Serratrice, 2009) stipulates that it would be harder to reach native-like processing of structures that are at the interface between syntax and other cognitive domains, such as the interface between syntax and discourse/pragmatics (in this theory, the latter are considered as external cognitive domains, being external to core generativist linguistic processes) as opposed to structures at interfaces within the language system, such as the syntax-semantics interface. In sum, both hypotheses claim that structures at the syntax-semantics interface should not cause any specific problems and should be easily processed in a manner similar to native speakers, which is not in keeping with the observed difficult L2 acquisition of VPCs (Laufer, 1997; Neagu, 2007).

### Lexical Knowledge of Verb Particle Constructions in L2

The challenge is even bigger when an L2 learner has to process structures that are not present in his or her native language, as is the case for French native speakers with VPCs, because they cannot benefit from any transfer from their French native language. However, using the exact same tasks for French–English bilinguals as Gonnerman and Hayes (2005) used for monolingual native speakers, Blais and Gonnerman (2013) showed that French–English bilinguals can develop both an explicit and implicit understanding of the semantic properties of VPCs, which approximate the sensitivity of native speakers as English proficiency increases. Indeed, their explicit similarity ratings became more correlated with those of monolingual native speakers as their English proficiency increased and the facilitation shown in the masked priming task was similar to that of native speakers. Thus, French–English bilinguals can succeed to master knowledge of structures that are not always semantically transparent and that are absent from their native language. Nonetheless, another major challenge is to integrate these VPCs in real time when reading a sentence, handling the various linguistic factors influencing L1 processing: the two possible particle positions, varying VPC dependencies and NP lengths.

### Theories of Sentence Processing in L2: Do Parsing Strategies Differ?

Bilingual sentence processing has noticeable differences from native processing (see Juffs and Rodriguez, 2014). Reading experiments using sensitive reaction time measures have shown that even highly proficient adult L2 speakers can differ from native speakers when processing difficult syntactic structures such as relative clauses (Frenck-Mestre, 2002; Hopp, 2014) or long-distance wh-dependencies (Cunnings et al., 2010; Dussias and Piñar, 2010; Felser et al., 2012). Two main recent approaches of L2 sentence processing provide different explanations for the observed differences between L1 and L2 processing.

First, according to the Shallow Structure Hypothesis (Clahsen and Felser, 2006a,b), L2 learners rely on different parsing mechanisms than native speakers and therefore we should observe differences in sentence processing even at the highest levels of L2 proficiency. Adult L2 learners would tend to process sentences making efficient use of non-grammatical information (such as semantic, pragmatic, probabilistic, or surface-level information) rather than building and manipulating abstract syntactic representations in real time (Clahsen and Felser, 2018), which would be the default processing pattern for native speakers. This tendency to underuse detailed syntactic analysis would hinder L2 learners’ grammatical parsing skills from becoming easily automatized and should be reflected in their processing of complex grammatical structures, even for the most proficient L2 learners.

In contrast, the other approach, the Fundamental Identity Hypothesis, states that L2 sentence processing is fundamentally similar to that used by native monolinguals and suggests that the observed differences in behavioral outcomes may be explained by processing difficulties resulting from less efficient lexical processing on one hand, and reduced working memory capacity on the other hand (Hopp, 2007, 2018).

Following Tily et al. (2010), Hopp (2016) explored the temporal dynamics of lexical access and syntactic integration in L1 and L2 processing and demonstrated the existence of a lexical processing difficulty in L2. In a self-paced reading experiment, the frequency of the verb was manipulated in sentences containing subject clefts or more structurally complex object clefts. Native English speakers and German–English bilinguals read subject clefts faster than object clefts. Processing sentences with object cleft structures became more difficult – relative to sentences with subject clefts structures – in the post-cleft region for verbs of lower frequency, while the difficulty was in the cleft region for verbs of higher frequencies. Critically, bilinguals showed post-cleft reading slowdowns, reflecting word frequency effects on structure building, for verbs of higher frequency than native speakers. These results support the existence of slower and less automatic lexical processing in L2 that could prevent bilinguals from displaying native-like syntactic processing. This slower lexical processing would itself result from weaker lexical links as well as from more diffuse lexical activation and representations. Hopp formalizes this phenomenon in the Lexical Bottleneck Hypothesis (Hopp, 2018), stating that bilinguals’ failure to demonstrate native-like syntactic processing does not necessarily imply faulty access to L2 grammar representations or a L2 parser that would be different from the L1 parser. The non-native-like syntactic processing instead originates from slower L2 lexical processing that would overload the limited L2 processing capacities, which can delay or prevent the building of a complete syntactic structure.

The second source of processing difficulties lies in the limited working memory capacity in L2. Working memory (WM) can be defined as a limited-capacity system to temporarily process and store information. Beyond this basic definition, little consensus exists on how to conceptualize, operationalize, and measure WM (Alptekin and Erçetin, 2009). For example, there is still no agreement on whether WM is language general (Just and Carpenter, 1992; Lewis et al., 2006) or if there are several subdomain-specific WM capacities (Caplan and Waters, 2013). There is no consensus either on whether WM is a fixed pool of independent resources or if it actually indexes language experience and biological factors (MacDonald and Christiansen, 2002; see Tan et al., 2017, for a comparison of several working memory models and how they account for individual differences for sentence processing at the interface of syntax and semantics).

Despite these theoretical divergences, differences in working memory measures have been robustly linked to individual differences in real-time L1 sentence processing, especially for the processing of complex structures or long-distance dependencies (see Engle, 2002; Farmer et al., 2012). On the other hand, the effect of working memory measures on L2 acquisition and processing has been much more controversial (see Juffs, 2004; Wen et al., 2015). McDonald (2006) showed that bilinguals’ grammaticality judgements were affected by low WM capacity, insufficient decoding abilities and slower processing speeds compared to native speakers. Results of studies looking at the effect of working memory on L2 processing of long-distance dependencies, such as ambiguous relative clauses, have been inconsistent (for a review see Juffs and Harrington, 2011; Williams, 2012). Some studies have found more native-like performance only for bilinguals with high working memory capacity (Frenck-Mestre, 2002; Dussias, 2003; Kim and Christianson, 2017), while other studies have found no effects of working memory on processing (e.g., Felser et al., 2003; Papadopoulou and Clahsen, 2003). Roberts (2012) argues that WM effects are limited to studies that include a metalinguistic task in their experimental design, such as grammatical judgements, while no WM effect should be observed in L2 processing for simple sentence comprehension.

However, in a meta-analysis of data from 79 samples involving 3,707 participants providing 748 effect sizes, Linck et al. (2014) found that working memory is positively associated with both L2 processing and proficiency outcomes, with an estimated population effect size (ρ) of 0.255. In addition, larger effect sizes of WM were found for verbal measures of WM (vs. non-verbal), for the executive attention control component of WM (vs. the storage component), that is best measured by complex WM tasks – such as reading span or operation span – than by simple WM tasks – such as digit or word span – and when WM was assessed in L2 rather than in L1.

Finally, Hopp (2014) reevaluated the effect of WM on relative clause attachment preferences using the same material and the same reading span task as in previous experiments that did not elicit a working memory effect on attachment preferences. However, the experimental design was slightly adjusted: a measure of automaticity of lexical processing was added, proficiency differences were controlled, more participants were tested than in previous experiments and WM was coded as a continuous variable, and not simply into high and low categories. Results showed that only bilinguals with high lexical automaticity and high working memory displayed native-like attachment preferences. This finding highlights the importance of controlling very precisely for a combination of individual variables that can impact L2 online processing of more complex structures, namely proficiency, lexical processing and working memory.

In sum, according to the first approach, the Shallow Structure Hypothesis, L2 sentence processing would be qualitatively different than L1 processing. It would only build shallow syntactic structures relying on semantic and pragmatic information. In the second approach, the Fundamental Identity Hypothesis, bilinguals’ limited resources regarding L2 lexical processing or working memory would underlie quantitative differences in otherwise fundamentally similar processing mechanisms. We now turn to previous results on L2 online processing of VPC in sentence contexts.

### L2 Processing of Verb Particle Constructions in Sentence Contexts

Matlock and Heredia (2002) analyzed differences in reading times between sentences with VPCs or with verb + preposition constructions (e.g., *He pulled the bank robbery off last week* vs. *He pulled the stained cloth off the table*) for bilinguals with various native languages and different ages of acquisition. In their experiment, only early bilinguals, like monolinguals, accessed VPCs more quickly than verb + preposition constructions. However, only the total reading time of the sentences was analyzed and the participants had a wide range of native languages, some of which included VPCs. Moreover, as the study did not specifically target variables impacting phrasal verb processing, VPC transparency was not controlled and only one NP length was used (3 words).

Schunack (2016) investigated particle placement preferences in Norwegian with native speakers and German–Norwegian bilinguals, who have structures with particles quite similar to VPCs in their German L1. Among other experimental conditions, participants read sentences including VPCs with adjacent or shifted particles and either 1-word or 4-word NPs. In an acceptability task, both native speakers and bilinguals showed a preference for adjacent over shifted placement of the particle. In a self-paced reading task, only native speakers displayed a preference for adjacent particles, reflected by faster reading times. Bilinguals displayed no preference, but showed larger variability in reading times than native speakers. This difference between offline preferences and online processing might point toward different processing demands in native and L2 speakers. However, no effect of proficiency, Norwegian VPC knowledge, working memory or other inter-individual variable was explored in this study.

### Present Study and Hypotheses

The present study looks at the processing of VPCs in a sentence context by bilinguals who do not have such structures in their L1. It replicates the experimental paradigm used in Gonnerman and Hayes (2005) in order to evaluate if bilingual processing follows the principles of syntactic and semantic domain minimization for different configurations of particle position, NP length and VPC dependency. If French–English bilinguals’ reading patterns are influenced in the same manner by factors that influence monolingual VPC processing, we will conclude that the processing of the VPC semantic dependency across the different syntactic configurations is similar in L1 and L2. We will especially evaluate if bilinguals have a preference for adjacent particles when the VPC dependency or the NP length increase.

Given the difficulty associated with VPCs in L2 and the variability observed in previous studies investigating L2 processing, we do not expect to find a single reading time pattern that would be representative of VPC processing for every bilingual. The role of specific VPC lexical knowledge and working memory capacity will be investigated as they are expected to modulate L2 VPC processing. Indeed, native speakers’ reading time patterns reflect VPC semantic dependency variation. Therefore, good knowledge of VPC meanings should be a necessary condition to display native-like processing. Moreover, reading VPCs in a sentence requires the rapid integration of three different elements (verb, particle, NP) with varying word orders and semantic dependencies, which requires a certain amount of processing capacity. We therefore expect working memory capacity to influence reading time patterns when the VPC semantic dependency is fully processed.

Given their limited knowledge of VPCs’ meaning, bilinguals with poor VPC lexical knowledge might display more shallow processing of VPCs. Consequently, they are expected to show no or only a weak effect of VPC dependency, and no clear preference for adjacent particles when the dependency is high. If VPC structures are not fully processed during sentence processing, this might reduce the need to minimize VP domain, as they could build a verbal phrase with only the verb and the particle. This would imply no specific preference for adjacent particles after long NPs. Finally, if the lack of VPC lexical knowledge results in no strong effects of the three linguistic variables, we do not expect working memory capacity to modulate reading patterns for these bilinguals.

On the contrary, participants with good VPC lexical knowledge are expected to fully process VPCs’ meaning and structure, including VPCs’ semantic dependency. As this would require the real-time integration of the verb, the particle, and the NP, we expect processing capacities indexed by WM to modulate reading time patterns. When a particle is adjacent, the reader has to combine the verb and particle first, access VPC meaning, and then immediately read and integrate the NP. If the reader has a very good lexical representation of the VPC or good processing capacities, adjacent particles will be preferred when they reduce different semantic and syntactic domains. Otherwise, processing the meaning of the VPC first might overload readers with more limited processing capacities, especially when this meaning is less compositional as VPC dependency increases. These readers would then display a slowdown in reading that might even spill over to the reading of the following NP, which would reduce the advantage of adjacent particles when the VPC dependency is high. Indeed, processing the longer semantic and syntactic domain associated with shifted particles might be less costly than the upfront lexical processing induced by adjacent particles. Therefore, we do not expect to find the native speakers’ preference for adjacent particles in speakers with more limited processing capacities.

We expect to observe most native-like patterns for participants with good lexical VPC knowledge and high WM capacity. These bilinguals should integrate the verb and the particle and access the VPC meaning more easily than any other bilingual. Following the different principles mentioned in Lohse et al. (2004), these bilinguals should prefer adjacent particles when the NP is long or when the VPC dependency is high. For low dependency VPCs, we expect to observe no preference between the two particle positions, or a preference for shifted particles for head-final NPs if these bilinguals are sensitive to the Particle-NP head dependency.

Finally, our results can be interpreted in light of the two theories of L2 processing. If bilinguals’ reading patterns are not modulated by working memory capacity and if even the most proficient bilinguals show no similar sensitivity to the factors that influence native speakers’ processing, this would support the view of a bilingual sentence processing using qualitatively different mechanisms and potentially more shallow structures than in native sentence processing. On the contrary, if our hypotheses are confirmed and bilinguals’ VPC lexical knowledge and WM capacity modulate bilinguals’ reading patterns, this would support theories situating the sentence processing difference between L1 and L2 in lexical processing efficiency as well as in available cognitive resources.

## Materials and Methods

### Participants

A group of 30 French–English bilingual speakers were recruited through internet and poster advertisements from the student population at McGill University (Montreal, Canada). These participants also took part in the first experiment reported in Blais and Gonnerman (2013). They received monetary compensation and were required to consider themselves non-native speakers of English but to have functional proficiency in English. None reported any neurological or language disorder, and vision was normal or corrected-to-normal. Three participants were excluded from our analysis due to experimental problems during the self-paced reading task. Therefore, the data from a total of 27 bilingual speakers (19 women), aged between 20 and 35 years old (*M* = 25.07, *SD* = 4.26), are reported in this study (**Table 2**). Their first exposure to English varied between 1 and 15 years old (*M* = 7.84, *SD* = 3.69). Participants’ English language proficiency was assessed through a written cloze task where they supplied the missing words in a text from a set of multiple-choice answers (cloze-test). Missing words in the text included content nouns and verbs as well as articles, prepositions, auxiliaries and verb conjugations. This test was adapted from a normed proficiency assessment tool developed at the University of Ottawa (University of Ottawa, 2010). Responses to the cloze task were scored out of a possible 30 points. Scores ranged from 10 to 30, with a mean of 24.3 and a standard deviation of 4.5. Participants’ working memory was assessed using a Reading Span test (Daneman and Carpenter, 1980). Scores ranged between 1.5 and 6 (*M* = 2.63, *SD* = 0.88).

**Table 2:** Participants’ characteristics (*n* = 27).

	Age (years)	Age of First exposure (years)	Cloze test score (maximum 30)	VPC semantic knowledge index	Reading Span Test (maximum 6)
Mean	25.07	7.84	24.3	0.38	2.63
*SD*	4.26	3.69	4.5	0.15	0.88
Range	20–35	1–15	10–30	–0.002–0.64	1.5–6

As reported in greater detail in Experiment 1 in Blais and Gonnerman (2013), each participant rated 35 or 36 items out of the 212 verb–particle constructions that were rated by monolinguals in Gonnerman and Hayes’ (2005) study. Participants were asked to rate the similarity in meaning of verb–particle/verb pairs on a scale from 1 (very dissimilar) to 9 (very similar). For each individual participant, the correlation between his or her ratings and the average ratings of the same items from monolinguals was calculated. In this study, we use this correlation score as an index of their VPC semantic knowledge. The correlations ranged from -0.002 to 0.64 with an average of 0.38 (*SD* = 0.15).

### Stimuli

Target stimuli for this experiment are an adaptation of the materials used in Gonnerman and Hayes (2005) and were built using the same 78 verb-particles that reflected uniformly the whole range of VPC dependencies (assessed through monolinguals’ similarity judgments). Six sentences were created for each verb-particle construction (VPC), reflecting the three direct object NP lengths and two particle positions (see **Table 3** for a sample set of sentences). To ensure that the task would not be too difficult for bilingual participants and avoid ceiling effects, given bilinguals’ more limited processing capacities compared to monolinguals (McDonald, 2006), NP lengths were reduced from previous material: short direct object NPs included two words (no change), medium NPs included three words (instead of five), and long NPs included five words (instead of nine). Two versions of each sentence were created for each VPC and each NP length, one with the verb and particle adjacent, and one with the particle placed after the direct object NP.

**Table 3:** Sample of stimuli for a VPC with medium dependency.

NP length	Particle position	Sample sentences
Short	Adjacent	The man will *look up* the word in the dictionary tonight
Short	Shifted	The man will *look* the word *up* in the dictionary tonight
Medium	Adjacent	The man will *look up* the unusual word in the dictionary tonight
Medium	Shifted	The man will *look* the unusual word *up* in the dictionary tonight
Long	Adjacent	The man will *look up* the origin of the word in the dictionary tonight
Long	Shifted	The man will *look* the origin of the word *up* in the dictionary tonight

Overall a total of 468 target sentences were created and divided into six lists, such that participants only saw one sentence for each VPC, that is, 78 total target sentences. The NP lengths and particle positions were balanced within each list.

Each sentence began with a two-word subject NP (e.g., *the boy*; *the driver*) that was controlled for frequency across conditions. All of the verb phrases were in the future tense (e.g., *The man will look up*…) in order to avoid irregular conjugations, and any supplementary morphological processing. Finally, plausible phrases were added to the end of the sentences to prevent wrap-up effects on the last target word of each sentence, for example, ‘*next month’* or *‘quickly’*.

### Procedure

In a single session, participants performed a battery of measures including a language background questionnaire, proficiency tests, a reading span test (Daneman and Carpenter, 1980) in English to assess working memory in the target language (see Alptekin and Erçetin, 2011; Hopp, 2014), a similarity judgment task [detailed results can be found in Blais and Gonnerman (2013)] and a self-paced reading task. The self-paced reading task was conducted before or after the similarity judgment task and tests of proficiency and memory, with the order of the different tasks counterbalanced across participants. Participants were tested individually in a sound-attenuated room. Sentences were presented one word at a time on a computer screen with black letters on a white background. Participants read at their own pace, pressing a button to replace the word they had just read with dashes and to display the next word of the sentence. Participants were given 4 practice sentences before reading the 156 experimental sentences (78 experimental sentences containing a VPC and 78 filler sentences in a random order). After 26 of the 78 target sentences, participants answered a yes-no content question to ensure careful reading and comprehension. Reading times for each button press were recorded.

## Results

### Preprocessing and Statistical Analyses

Data were analyzed with mixed effects regression modeling using R (R Development Core Team, 2017) and the lme4 package (Bates et al., 2015). Bilinguals were on average 82.4% (*SD* = 5.4%) accurate in answering the comprehension questions. Three participants who scored below the exclusion cutoff of 75% accuracy were removed from the subsequent analyses. Moreover, reading times for sentences where participants did not answer the comprehension question correctly were excluded from analysis, resulting in a loss of 5.3% of reading times. Finally, reading times that were below 100 ms or above 3500 ms, or outside three standard deviations of a participant’s mean reading time were also removed, resulting in a loss of 1.9% of remaining reading times.

Predictors of interest in the models were Particle position (adjacent or shifted) that was sum-coded, VPC Dependency (based on the reverse of monolinguals’ mean similarity ratings, with values from 1, for low dependency like *finish up*, to 9 for high dependency like *chew out*), NP length (2, 3, or 5 words), in addition to their interaction with working memory, as measured by the reading span test, and with VPC lexical knowledge, assessed through the correlation of each bilingual’s similarity ratings with the monolinguals’ mean ratings. The interaction among those 5 variables was computed, as well as all other 2, 3, or 4-way possible interactions.

Proficiency, measured by the results of a cloze score test, covaries only minimally with bilinguals’ VPC lexical knowledge (*r* = 0.25, *p* < 0.05). It has been added to the model in order to control for potential specific proficiency effects, independent of VPC lexical knowledge and working memory. Working memory scores also covary minimally with proficiency (*r* = 0.17, *p* < 0.05). Finally, an extremely weak correlation was found between VPC lexical knowledge and Working memory (*r* = 0.03, *p* < 0.05).

We also assessed collinearity between proficiency, VPC lexical knowledge and working memory using Baayen’s condition number criterion (Baayen, 2008, p. 200). A condition number of 19.82 was found between proficiency and VPC lexical knowledge while it was equal to 19.73 between proficiency and working memory and had a value of 10.27 between VPC lexical knowledge and working memory. These three condition numbers reflect medium collinearities between the three variables – defined as having a value ‘*around 15*’ – but are not associated with potentially harmful collinearities – defined as having values of 30 or more.

Models were fit using the maximal random effect structure justified by the design that would converge on the data (Barr et al., 2013). This included random intercepts for VPC and participant, in addition to ‘by participant’ random slopes for Particle position, VPC Dependency, NP length, as well as for interactions between Particle position and VPC Dependency, Particle position and NP length, VPC Dependency and NP length. Finally, the model also included ‘by VPC’ random slopes for Proficiency, Working memory, VPC lexical knowledge and the interaction between Working memory and VPC lexical knowledge. Reading times were log transformed and all continuous predictors were centered and normalized.

### Reading Times for the Critical Region

A model was run on the critical region, defined as including reading times for verbs, noun phrases and particles (**Table 4**). This model found no main effect of Particle position (β = 0.005, *t* = 0.96), NP length (β = 0.007, *t* = 1.48), Working memory (β = 0.007, *t* = 0.22), or VPC knowledge (β = -0.009, *t* = -0.29). There was a nearly significant main effect of VPC dependency, with lower RTs for higher dependency VPCs (β = -0.011, *t* = -1.71). Finally, there was a strong main effect of proficiency with more proficient participants reading significantly faster (β = -0.123, *t* = -4.88).

**Table 4:** Statistical model for reading times at the region of interest.

Predictors	Parameter estimates		
	β	*SE*	*t*
Intercept	2.594	0.016	167.32
Particle position	0.005	0.005	0.96
NP length	0.007	0.004	1.48
VPC dependency	–0.011	0.007	–1.71
Working memory	0.007	0.031	0.22
VPC lexical knowledge	–0.009	0.032	–0.29
Proficiency	–0.123	0.025	–4.88
Particle position × NP length	0.002	0.010	0.21
Particle position × VPC dependency	–0.009	0.008	–1.10
NP length × VPC dependency	0.011	0.008	1.48
Particle position × Working memory	0.006	0.011	0.51
NP length × Working memory	0.008	0.009	0.85
VPC dependency × Working memory	0.001	0.012	0.12
Particle position × VPC lexical knowledge	–0.003	0.011	–0.24
NP length × VPC lexical knowledge	–0.005	0.009	–0.52
VPC dependency × VPC lexical knowledge	–0.005	0.012	–0.44
Working memory × VPC lexical knowledge	–0.109	0.07	–1.60
Particle position × NP length × VPC dependency	–0.009	0.011	–0.84
Particle position × NP length × Working memory	–0.012	0.019	–0.60
Particle position × VPC dependency × Working memory	–0.027	0.016	–1.69
NP length × VPC dependency × Working memory	–0.008	0.015	–0.55
Particle position × NP length × VPC knowledge	0.034	0.020	1.72
Particle position × VPC dependency × VPC knowledge	0.012	0.016	0.72
NP length × VPC dependency × VPC knowledge	0.010	0.016	0.67
Particle position × Working memory × VPC knowledge	0.005	0.024	0.19
NP length × Working memory × VPC knowledge	–0.004	0.0200	–0.22
VPC dependency × Working memory × VPC knowledge	0.031	0.025	1.24
Particle position × NP length × VPC dependency × Working memory	0.040	0.021	1.85
Particle position × NP length × VPC dependency × VPC knowledge	–0.069	0.023	–3.03
Particle position × NP length × Working memory × VPC knowledge	0.023	0.044	0.54
Particle position × VPC dependency × Working memory × VPC knowledge	0.015	0.037	0.39
NP length × VPC dependency × Working memory × VPC knowledge	0.031	0.035	0.91
Particle position × NP length × VPC dependency × Working memory × VPC knowledge	0.145	0.050	2.87

**FIGURE 1:**
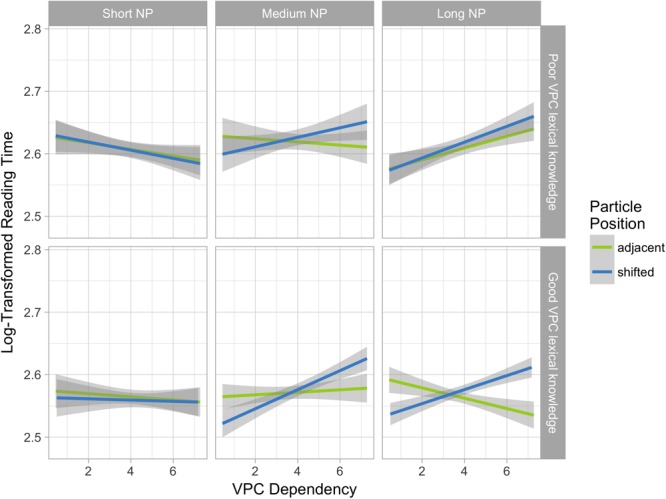
Interaction among the three linguistic variables (Particle position, VPC dependency, and NP length) and VPC lexical knowledge. VPC lexical knowledge groups were created using a median split for illustrative purposes only. Statistical models were run using the continuous variable.

The model revealed a significant interaction between Particle position, VPC dependency, NP length and VPC Knowledge (β = -0.069, *t* = -3.03), as can be seen in **Figure 1**. An interaction between Particle position and VPC Dependency appears only for sentences with long NPs in participants with good VPC lexical knowledge: when Dependency increases, RTs for sentences with shifted particles increase, while they decrease for sentences with adjacent particles. No such interaction is found for participants with poor VPC lexical knowledge. For sentences with medium NPs, there seems to be a trend toward the same interaction for both bilinguals with poor and good lexical knowledge. For sentences with short NPs, there is no significant VPC Dependency or Particle Position effect, nor an interaction between them for any subgroup.

**FIGURE 2:**
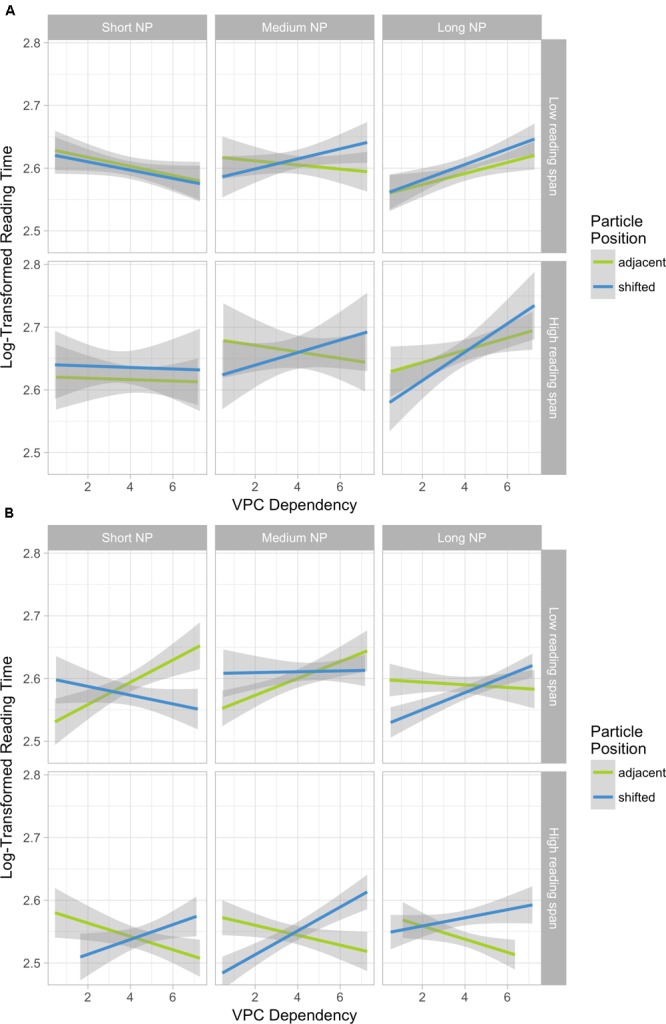
Interaction among the three linguistic variables (Particle position, VPC dependency and NP length), VPC lexical knowledge and Working memory. VPC lexical knowledge and Working memory groups were created using a median split for illustrative purposes only. Statistical models were run using the continuous variables. Ribbons were derived from the geom_smooth function in ggplot R package. **(A)** Participants with Poor VPC lexical knowledge. **(B)** Participants with Good VPC lexical knowledge.

Finally, there was a significant 5-way interaction among Particle Position, Dependency, NP length, VPC Knowledge and Working memory (β = 0.145, *t* = 2.87) as illustrated in **Figure 2**. There was no significant Particle Position by Dependency by NP length interaction in any subgroup of participants with poor VPC lexical knowledge (**Figure 2A**). We can only observe a trend toward a Dependency by NP length interaction, with a more pronounced Dependency effect for longer NPs.

In contrast, bilinguals with good lexical knowledge present a different pattern of Particle Position by Dependency interaction according to their working memory capacity (**Figure 2B**). For sentences with short NPs, we can see that the absence of any effect observed for the whole group of participants with good lexical knowledge in the previous 4-way interaction was in fact the result of two opposite interactions between VPC dependency and position for each working memory subgroup. Thus, for the good lexical knowledge and low working memory (LWM) subgroup, RTs increase when the VPC dependency increases for adjacent particles, while the RTs decrease for shifted particles, meaning that these bilinguals would read *He will screw the painting up* more quickly than *He will finish the painting up*. On the contrary, for the good lexical knowledge and high working memory (HWM) subgroup, RTs increase when the VPCs are more opaque only for shifted particles, while the RTs decrease for adjacent ones: they read *He will finish the painting up* more quickly than *He will screw up the painting*, which is what is observed for monolinguals. They also tend to read the shifted particle sentences more quickly than adjacent ones when the dependency is low, which is exactly what is predicted by Lohse et al. (2004). For sentences with medium NPs, both LWM and HWM subgroups show interactions that are close to those described for sentences with short NPs.

The LWM subgroup’s RTs display, for sentences with long NPs, the interaction that is observed for sentences with short and medium NPs in the HWM subgroup: only RTs of sentences with shifted particles increase when VPC dependency gets higher, while being lower than RTs of sentences with adjacent particles when VPC dependency is low.

For sentences with long NPs, the HWM subgroup’s RTs increase with VPC dependency only for sentences with shifted particles, and there is no difference in the RTs between shifted and adjacent particles sentences when the VPCs are low dependency. These findings indicate that the NP length effect is stronger than the Position effect, a phenomenon also observed in monolinguals. Therefore, only the subgroup with good lexical knowledge and good working memory presents native-like patterns, highlighting the contribution of both variables.

## Discussion

To understand how French–English bilinguals use their grammatical and semantic knowledge of VPCs during real time sentence processing, we studied their reading time patterns when exposed to sentences including VPCs with different VPC dependencies, NP lengths and particle positions. Previous studies investigating native speakers’ VPC processing (Gonnerman and Hayes, 2005) found main effects of particle position, NP length and VPC dependency, with higher reading times for sentences that include shifted particles, long NPs and high dependency VPCs. More importantly, these factors interacted, leading to highest reading times for shifted particles with either long NPs or high dependency VPCs. These experimental results were in line with Lohse et al. (2004)’s principle of processing efficiency, suggesting that semantic or syntactic processing demands are reflected by different dependency domains that need to be minimized in order to facilitate VPC processing. Reading times reflected monolinguals’ particle placement preferences. First, adjacent particles are preferred when the object NP is long in order to minimize the VP syntactic domain. Second, adjacent particles are also preferred for high dependency VPC, as their high semantic dependency is accentuating the need to minimize VPC semantic dependency domain. Finally, there is no strong preference in particle placement in other cases; only a minor preference for shifted particles can be observed when the head of the NP is in final position, as it minimizes the Particle-NP Head semantic dependency.

In contrast with native speakers’ results, our analyses revealed no main effects of Particle position or NP length and only a marginally significant effect of VPC dependency. Therefore, when considering all bilinguals together, none of the linguistic factors seems to significantly influence VPC processing in a sentence context. This overall absence of effects can be expected from previous results found in Blais and Gonnerman (2013) if we consider that not all bilinguals have good VPC lexical knowledge, which would be necessary to fully process VPCs in order to elicit a VPC dependency effect as well as other associated effects. VPC lexical knowledge and working memory do not have a main effect on reading times, but they significantly modulate the interaction between NP length, particle position, and VPC dependency, leading to different reading patterns for different subgroups of participants.

First, bilinguals with poor VPC lexical knowledge do not seem to display any clear particle position preference across the different syntactic and semantic VPC configurations. Their lack of good VPC lexical knowledge prevents them from fully processing VPCs and their varying dependencies in a native-like way. The only noticeable effect is a VPC dependency effect in long NPs; regardless of the particle position, high dependency VPCs are read more slowly than low dependency VPCs. This effect is also found in native speakers for shifted particles. Despite these bilinguals’ lack of strong VPC lexical knowledge, the combination of increased semantic demands on one hand – long NPs and shifted particles increase the size of the VPC dependency domain, which is very costly for high dependency VPCs – and increased syntactic pressure on the other hand – long NPs extending the VP domain – might be strong enough to induce a VPC dependency effect resulting from weaker VPC lexical knowledge. However, for adjacent particles, increased reading times for high dependency VPCs – relative to low dependency VPC – are usually not observed in L1 VPC processing. We might speculate that the necessity to integrate a long NP after a high dependency VPC that might not be entirely understood given its more opaque meaning could be an explanation for the observed reading slowdown. In sum, bilinguals with poor VPC lexical knowledge display quite different reading patterns than native speakers, with no particle position preference and a limited effect of dependency for longer NPs.

Second, participants with good lexical knowledge but more limited working memory display a preference for shifted particles for two VPC configurations that is not found in native speakers. The first one concerns high dependency VPCs with short NPs. Native speakers would prefer adjacent particles because of the high dependency VPCs. Indeed, shifting the particle increases the VPC semantic domain and forces readers to process a potentially implausible NP before accessing the dependent particle. On the contrary, for that group of bilinguals, shifted particles are preferred: *The professor will chew the student out* is preferred over *The professor will chew out the student*. For them, it seems that the cost of extending the VPC semantic domain and the possibly associated implausible NPs is inferior to the cost of processing the meaning of high dependency VPCs online and immediately integrating the NP. High dependency VPCs might generate a processing overload that could persist during the processing of the NP. The second configuration in which these bilinguals prefer shifted particles, unlike native speakers, is for low dependency VPCs with long NPs. In this case, given the long NP, native speakers would prefer adjacent particles to minimize the VP domain. However, these bilinguals prefer *The student will finish the exciting project on bilingualism up* over *The student will finish up the exciting project on bilingualism*. Again processing the VPC upfront might lead to a larger processing overload than processing the shifted particle after a long NP that can be easily integrated, given the low dependency VPC. Lexical processing difficulties are more costly than a non-optimal syntactic processing (here a non-minimal syntactic VP domain). In all these cases, non-native reading patterns and particle placement preferences can be tied to a lack of processing capacities for efficiently combining the verb with the adjacent particle in order to access the VPC meaning, and immediately process and integrate the object NP. Lexical processing difficulties would cause a slower and more serial sentence processing, and preclude native-like syntactic-driven particle placement preferences for bilinguals with good VPC lexical knowledge but limited processing capacities.

Finally, participants with good lexical knowledge and high WM display the most native-like reading patterns. They show a preference for adjacent particles both when the verb dependency is high and when the NP is long. Short NP lengths and low VPC dependencies generally do not favor a specific particle placement, and a preference can be driven by the position of the head of the NP. In our experiment, the 2- or 3-word NPs were almost all head final with the structure article + noun, such as ‘*the project,’* or article + adjective + noun, such as ‘*the new project,’* while only half of the long NPs had their head as the final word. Therefore, the preference for shifted particles observed for these bilinguals for short NP lengths and low VPC dependencies reflects the minimization of the Particle-NP head domain. It also allows them to access all main semantic elements of the sentence earlier.

The main difference between monolinguals and bilinguals with good lexical knowledge and high working memory is that the latter seem to show no main effects of Particle position or VPC dependency. Otherwise, reading dynamics are in keeping with those observed in monolinguals (Gonnerman and Hayes, 2005; Gonnerman, 2012). They reflect the tension between syntactic and semantic domain minimizations in different configurations of particle position, NP length and VPC dependencies, which is at the core of the VPC processing efficiency principle proposed by Lohse et al. (2004).

As other structures with non-literal meaning such as idioms, VPCs are multi-word elements that cause lexical difficulties and are hard to acquire and master in a second language. Indeed, understanding a VPC is not straightforward for multiple reasons. First, two words must be combined to access VPC meaning and are sometimes separated by an NP. Second, the compositionality of VPC meaning varies. Third, VPCs can be polysemous. Finally, retrieving VPC meaning can be highly susceptible to interference, given that a verb can often be associated with many particles. Therefore, bilinguals’ lexical knowledge might rely on less entrenched and weaker lexical representations than in L1, especially for the non-compositional high dependency VPCs.

However, good VPC lexical knowledge is necessary to develop any particle position preference when processing VPCs in real time in sentence contexts. The complex VPC semantic structure highlights the importance of lexical knowledge in sentence processing. Meaning is often considered as easy and ‘*coming for free*’ in L2 (Slabakova, 2008), but our results show that lexical knowledge deficits can hinder further syntactic processing and might be a bottleneck in some aspects of second language sentence processing.

However, to process VPCs using the principles observed in native processing, good lexical knowledge is not enough and bilinguals also need enough processing capacity. Indeed, VPCs appear in different syntactic configurations, which makes their parsing less predictable. Moreover, each particle position in the different configurations of NP lengths and VPC dependencies favors semantic and syntactic dependencies in different ways, so there is no possible default processing strategy. In our study, this online processing capacity was measured by a reading span task, a complex WM task more likely to reflect the attentional control component of WM than only storage capacity (Linck et al., 2014); (see Alptekin and Erçetin, 2009, for a discussion on the excessive focus on the storage aspect of WM in L2 to the detriment of the processing component). Indeed, when processing VPCs, readers do not need to keep a word or a constituent in memory for a long period of time, as the different VPC dependencies are rather short. Rather, they need to coordinate syntactic and semantic information very efficiently in real time. VPCs are multi-word expressions and the reader needs to combine its two words before accessing its meaning, and integrating it with other sentence constituents. Thus, processing VPCs requires not only lexical processing efficiency but also combinatorial efficiency. It is therefore not surprising that a WM measure, treated as a continuous variable, captured individual differences in VPC processing in bilinguals, without asking our participants to perform a metalinguistic task (contra Roberts, 2012). Therefore, it might be prudent, when studying VPC processing in L2, and more generally sentence processing, to include individual measures of proficiency, lexical knowledge and processing capacities to not miss different processing patterns that might be hidden in the overall average.

We can conclude that good VPC lexical knowledge is necessary but not sufficient to yield native-like processing of VPCs in a sentential context. Indeed, a high level of working memory is also necessary in order to process the non-compositional meaning of VPCs efficiently and combine it with the object NP. Our findings support theories highlighting the importance of lexical processing in L2 sentence processing, along with individual variability in processing capacities, as indexed here by working memory.

The Shallow Structure Hypothesis (SSH) postulates the use of different representations and mechanisms than in L1 processing even for the most proficient bilinguals. However, in our study bilinguals with good lexical knowledge and good processing capacities displayed native-like semantic and syntactic preferences regarding particle placement. Some could argue that these preferences could simply reflect the probabilistic distribution of particle placement observed in received input, but this interpretation would not explain why the reading time patterns differ as a function of working memory level. Therefore, the SSH does not seem compatible with our findings as it cannot account for native-like syntactic processing for some bilinguals, nor for the observed difference between bilinguals with different levels of working memory.

Throughout our study we have been assessing if bilinguals’ VPC processing is ‘native-like.’ Doing so, we do not want to imply that bilinguals’ ultimate endpoint should be to process their second language as monolinguals process their native language. First, bilinguals are not two monolinguals in one person (Grosjean, 1989). Second, some variability exists in language processing across native speakers and the ideal representative native speaker does not exist. Finally, bilinguals could follow different trajectories building on their two languages and could also go “beyond the native speakers” (Cook, 1999). We compare bilinguals to monolingual native speakers because they remain a natural and relevant reference group: they have extensive experience with English and as little interference with another language as possible. Moreover, the theoretical and processing formalisms that have been established in native processing, both through corpus studies and experiments, provide a framework for the analysis of our results. Beyond the simple ‘native-likeness,’ we aimed to understand how bilinguals process VPCs and what variables can predict different processing across individuals.

Additional studies should be conducted to confirm our results with a larger sample of participants while also controlling for the nature and evolution of L2 exposure. Moreover, using this paradigm with participants from various native languages, some of which have VPCs, would reveal potential effects of L1 transfer effects. It would also be interesting, both for L1 and L2 processing, to manipulate systematically the position of the head of the NP and the NP plausibility, as well as to include verb + preposition constructions in the experimental paradigm to contrast VPC processing with the processing of this similar construction. Finally, differences in processing capacities are not just thought to reflect differences between L1 and L2 processing, but should also explain the variability that can be observed in L1 processing. However, in previous studies investigating VPC processing, native speakers have always been considered as one uniform group. It would be valuable to explore the inter-individual variability in monolinguals and to assess potential effects of working memory on reading dynamics.

## Conclusion

The results of our self-paced reading experiment targeting VPCs show that native-like online processing of unfamiliar structures at the interface of syntax and semantics is possible. These findings are in keeping with second language processing that is fundamentally similar to native processing but hindered by difficulties in lexical acquisition and processing as well as by limited processing capacities.

## Ethics Statement

This study was carried out in accordance with the guidelines of the Institutional Review Board (IRB) of the McGill University Faculty of Medicine, which also approved the protocol. All subjects gave written informed consent before beginning the experiment in accordance with the Declaration of Helsinki.

## Author Contributions

LG designed the study, created the stimuli, and carried out the experiments. Supervised by LG and SB, AH pre-processed the data and ran the statistical models in R. All authors contributed to data interpretation. AH wrote the final manuscript that LG and SB critically revised. All authors listed have made a substantial, direct and intellectual contribution to the work, and approved it for publication.

## Conflict of Interest Statement

The authors declare that the research was conducted in the absence of any commercial or financial relationships that could be construed as a potential conflict of interest.
